# Nutrient content of some Cameroonian traditional dishes and their potential contribution to dietary reference intakes

**DOI:** 10.1002/fsn3.334

**Published:** 2016-01-20

**Authors:** Roger Ponka, Elie Fokou, Eric Beaucher, Michel Piot, Frédéric Gaucheron

**Affiliations:** ^1^ Department of AgricultureLivestock and Derivated ProductsThe Higher Institute of the SahelUniversity of MarouaMarouaCameroon; ^2^ Department of BiochemistryFaculty of ScienceUniversity of Yaoundé IYaoundéCameroon; ^3^ UMR 1253 Science et Technologie du Lait et de l'Œuf Inra‐Agrocampus RennesRennesFrance

**Keywords:** Amino acid composition, Cameroon, dietary reference intakes, minerals composition, Proximate composition, traditional dishes, Vitamin A activity composition

## Abstract

Malnutrition is a serious public health problem in Cameroon. The research study was conducted to determine nutrient content of some Cameroonian traditional dishes and their potential contribution to dietary reference intakes. These dishes were *Ekomba*, prepared from maize flour with roasted peanuts paste; *Ekwang*, prepared from crushed cocoyam tubers and cocoyam leaves; *Tenue militaire*, prepared from dried maize flour and cocoyam leaves and *Koki*, prepared from dried crushed cowpea seeds. The samples were subjected to proximate, minerals, carotenoids, and amino acids analyses. Results showed that the protein content ranged between 1.4 and 5.4 g/100 g edible portion. The mineral content expressed in mg/100 g edible portion ranged between 13.4 and 38.9 (calcium), 12.9–30.7 (magnesium), 336.2–567.9 (sodium), 63.3–182.7 (potassium), 0.5–1.5 (iron), 0.3–1.1 (zinc), 0.1–0.2 (copper), and 0.3–0.4 (manganese). Vitamin A activity content ranged between 0.1 and 0.4 mg Retinol Activity Equivalents/100 g edible portion. Consumption of each dish (100 g) (*Ekwang*,* Tenue militaire*, and *Koki*) by children aged 1–2 years would meet more than 100% of their daily recommended intake for vitamin A. Except in *Ekomba*, essential amino acids in all dishes represented up to 33% of total amino acids, indicating a good equilibrium between amino acids. This up‐to‐date appropriate information will contribute for the calculation of accurate energy and nutrient intakes, and can be used to encourage the consumption of these dishes.

## Introduction

Malnutrition, with its two constituents of protein–energy malnutrition and micronutrient deficiencies, continues to be a major health burden in developing countries (FAO 2010). It is globally the most important risk factor for illness and death, with hundreds of millions of pregnant women and young children particularly affected. Apart from marasmus and kwashiorkor (the two forms of protein–energy malnutrition), deficiencies in iron, iodine, vitamin A, and zinc are the main manifestations of malnutrition in developing countries (Brabin and Coulter 2003).

Malnutrition is the consequence of a range of factors that are often related to poor food quality, insufficient food intake, and severe infectious diseases, or most times, combinations of the three (Muller and Krawinke 2005). It is consequently the most important risk factor for the burden of disease in developing countries (Black 2003). It is the direct cause of about 3,00,000 deaths per year and is indirectly responsible for about half of all deaths in young children (Black et al. 2003).

In Cameroon, approximately 45,000 children die each year due to malnutrition (UNICEF Cameroon 2009). Despite sufficient quantity and diversity of food resources in Cameroon, malnutrition rates among children under 5 years have persisted and are on the rise (EDS‐MICS 2011). One of the contributing factors to malnutrition is the lack of nutritional and composition information of foods eaten in a given area. The energy and nutrient content of a food item provides information on its nutritional characteristics, and this information has through the years been captured in food composition databases around the world. Information on the energy and nutrient composition of foods is a prerequisite for analyzing dietary intake data and aids in establishing the diet–disease relationship in a community or country. In addition, information on the nutrient composition of food is also essential for achieving dietary intake goals (Burlingham 2003; Pennington 2008).

Because eating habits differ from one region to another in Cameroon, one of the effective approaches to fight against malnutrition is to make an inventory of food ready for consumption in each of the 10 regions of Cameroon and investigate their nutritional values. In this regard, some works were done on dishes of Littoral, Centre, West, and Far north regions of Cameroon (Kana et al. 2008; Fokou et al. 2009; Gimou et al. 2014; Ponka et al. 2015). However, food composition data for Cameroon are limited, both in terms of the number of foods and the nutrients listed. Furthermore, the food composition data contain mostly nutritional composition values for single‐item foods rather than composite dishes. Thus, the aim of this study was to determine nutrient content of some Cameroonian traditional dishes and their potential contribution to dietary reference intakes.

## Materials and Methods

### Design and sampling procedures

Among the women selling the traditional dishes to be studied, the highest sellers were selected to take part in the study. An appointment was scheduled at the home of the participants in order to observe the different steps of cooking, note the duration of cooking, identify the ingredients, and weigh the quantities used for the various dishes. These dishes were *Ekomba* (maize flour with roasted peanuts paste), prepared from dried maize grains and dried peanut seeds and consumed as a cake *Ekwang* (crushed cocoyam tuber with cocoyam leaves) is a compact paste prepared from cocoyam tubers and cocoyam leaves; *Tenue militaire* (Maize flour with cocoyam leaves), it is a dish prepared from dried maize grains and cocoyam leaves; *Koki* (crushed cowpea), it is a dish prepared from dried cowpea seeds and consumed with several complements such as tubers, bananas, and plantains. The local names, forms of the dishes, ingredients and proportions, as well as the scientific names of the basic ingredients are listed in Table 1.

**Table 1:** List of some traditional dishes consumed in Yaoundé‐Cameroon

Local name of the dish	Forms of the dish	Ingredients (%)	Scientific names of the basic ingredients
*Ekomba*	Bundle	Dried maize grains (58.2), dried peanut seeds (23.5), crayfish (2.1), salt (0.9), Crude palm oil (1.6), pepper (0.6), water (13.0)	*Zea mays, Arachis hypogea*
*Ekwang*	Compact paste	Cocoyam tubers (43.6), Cocoyam leaves (25.6), smoked fish (3.5), crayfish (1.7), water (17.8), pepper (0.4), crude palm oil (5.9), maggi cube (0.1), onion (1.6)	*Xanthosoma sp*.
*Tenue militaire*	Bundle	Dried maize grains (41.8), cocoyam leaves (30.7), salt (0.2), maggi cube (0.2), pepper (0.4), crude palm oil (6.9), crayfish (3.4), water (16.5)	*Zea mays, Xanthosoma sp*.
*Koki*	Bundle	Dried cowpea seeds (77.1), crude palm oil (5.6), water (15.4), pepper (1.4), salt (0.4)	*Vigna unguiculata*

### Preparation of dishes and collection

#### *Ekomba* (maize flour with roasted peanuts paste)

Dried maize grains were ground in a grinding mill. The flour obtained was sieved to eliminate the chaff. Some roasted peanuts were ground and added to the flour. The mixture was seasoned with pepper, salt, crude palm oil, and crayfish. A small quantity of water was added to the mixture to obtain a homogenous paste. The paste was wrapped in banana leaves. Some support was put in the pot containing water and the bundles of paste placed over it to prevent direct contact with the bundles and water. The pot was then cooked for 2 h under a vaporer.

#### *Ekwang* (crushed cocoyam tuber with cocoyam leaves)

Peeled cocoyam tubers were washed and crushed. Salt and water were added to the obtained paste. The latter was then wrapped in young cocoyam leaves cut into small rectangles. After boiling for about 40 min; all these were seasoned with crayfish, smoked fish, salt, peppers, maggi cubes, crude palm oil, and the whole mixture was kept boiling for about 1 h 30 min.

#### *Tenue militaire* (maize flour with cocoyam leaves)

Dried maize grains were ground in a grinding mill. The flour obtained was sieved to eliminate the chaff. The water containing washed chaff was added to the sieved flour. The young cocoyam leaves cleared were cut and added to the previous mixture. The latter was seasoned with pepper, salt, maggi cube, crude palm oil, and crayfish. The paste was then wrapped in banana leaves. Some support was put in a pot containing water and the bundles of paste placed over it to prevent direct contact with the bundles and water. The pot was then cooked for 4 h under vaporer.

#### *Koki* **(**crushed cowpea and palm oil)

Dried cowpea seeds previously soaked 30 min were rubbed vigorously to eliminate the chaff. The dehulled seeds combined with the peppers were then crushed. Salt, water, and slightly heated crude palm oil were added in the obtained paste. The mixture was then wrapped in banana leaves and then placed in a pot containing water and boiled for about 3 h. These dishes were selected based on their high frequency of consumption. Data on their nutritional composition are useful to evaluate their potential contribution to dietary reference intakes.

These samples were bought in the same quantity in such a manner as to have six samples per dish. A total of 24 analytical samples were collected and put into clean dry airtight sample containers. They were transported in dry ice to the laboratory. All samples were lyophilized and stored frozen at −20°C until analysis. However, the moisture content was determined on fresh samples.

### Proximate composition

The moisture content, ash, fat, protein, and dietary fiber of all the samples were determined according to the method of AOAC (2000). All samples were analyzed in triplicate. Total carbohydrate content was calculated by difference.

### Mineral determination

The mineral content (calcium, magnesium, sodium, potassium, iron copper, zinc and manganese) was determined according to the standard methods of the Association of Official Analytical Chemists AOAC (2005), using an atomic absorption spectrometer. The sample was ashed at 550°C and the ash boiled with 10 mL of 20% HCl in a beaker and then filtered into a 100 mL standard flask. All samples were analyzed in triplicate.

### Analysis of carotenoids

Carotenoids were extracted following the method described by Hart and Scott (1995). Approximately 5 g of sample was extracted with tetrahydrofuran/methanol (1:1, THF:MeOH) followed by petroleum ether (40–60°C) and antioxidant, and 0.1% butylated hydroxytoluene (BHT). The aqueous phase was extracted twice with petroleum ether and the washings were added together. Saponification was performed with addition of 40% KOH/MeOH to the extract, with a flow of nitrogen gas and was kept in the dark at room temperature for an hour. Carotenoid was extracted from the KOH/MeOH phase with petroleum ether and washed with distilled water until pH was neutral. The extract was dried by rotatory vacuum evaporation and was diluted again with petroleum ether and dichloromethane to a volume of 5 mL. Twenty *μ*L samples were injected into the reverse‐phase high performance liquid chromatography (HPLC) system for carotenoid analysis. Analysis was done in triplicate.

### High Performance liquid chromatography analysis of carotenoids

Quantitative analysis on the amount of carotenoids present was performed, using HPLC with a reverse phase column, Waters *μ*‐Bondapak C_18_ column (30 cm x 3.9 mm i.d., Waters, Milford, MA) operated at 30°C. The column was preceded by a Waters Guard‐Pak precolumn **(**Waters) module housing a disposable Guard‐Pak precolumn insert packed with the same material as that in the analytical column. A Waters 510 pump (Waters Corp. Milford, MA) was used to deliver the mobile phase which was a ternary mixture of acetonitrile, methanol, dichloromethane (MeCN: MeOH:DCM) 75:20:5 v/v/v, containing 0.1% BHT and 0.05% triethylamine. Individual carotenoid identification was based on the spectrum and the retention time of the pure substances. Known carotenoid standards (*β*‐carotene and  utein) were obtained from Sigma‐Aldrich (Saint‐Quentin‐Fallavier, France). The carotenoids of the samples were quantified (mg/100 g edible portion) by projection of their areas on the corresponding calibration curve. All determinations were run in triplicate.

### Vitamin A activity

The content of *β*‐carotene obtained was used to calculate the vitamin A activity in the sample. Conventionally, the nutritional significance of carotenoid is related to the provitamin A activity. Vitamin A activity of *β*‐carotene expressed as mg retinol activity equivalents (RAE)/100 g sample was calculated as RAE = (mg *β*‐carotene)/12 according to Institute of Medicine (2001).

### Amino acid determination

Amino acid analysis was carried out by ion‐exchange chromatography under the experimental conditions recommended for protein hydrolysates. Each sample containing 5.0 mg of protein was acid hydrolyzed with 1 mL of 6 N HCl at 110°C for 24 h in vacuum‐sealed glass tubes (Moore and Stein 1963; Davies and Thomas 1973). The sulfur‐containing amino acids were oxidixed using performic acid before 6 N HCl hydrolysis. The amino acid analysis of the hydrolysed samples was then carried out by ion‐exchange chromatography on a Biochrom 30 automatic amino acid analyzer (Biochrom Ltd, Cambridge, UK) according to Spackman et al. (1958) using lithium citrate buffers as eluants and ninhydrin postcolumn reaction system. The amino acids composition was calculated from the areas of standards obtained from the integrator and expressed as percentages of the total protein. All determinations were run in triplicate. Tryptophan was not determined.

### Evaluation of protein quality (amino acid score)

The amino acid score was calculated using the following equation:

$$\text{Amino acid score}\;=\;\frac{\text{mg of essential amino acid per g of test protein}}{\text{mg of the same essential amino acid per g of protein in requirement pattern}}\;\times \;100.$$

The requirement pattern suggested by the FAO/WHO/UNU (2007) for children of 1–2 years old was used for this purpose.

### Statistical analysis

Results of the composition of the sample were expressed as means ±SD of six samples with three replicates per variety. Data were analyzed by one‐way analysis of variance (ANOVA). The Statistical Package for Social Science (SPSS) version 16.0 (Nikiski, AR, USA) was used for data analysis. Differences between samples were tested according to Tukey test and considered to be significant when *P *<* *0.05.

## Results and Discussion

### Proximate, mineral, carotenoid, and vitamin A activity compositions

#### Proximate composition

The proximate composition of the dishes is presented in Table 2. The moisture content in the samples ranged from 61.5 for *Ekomba* to 77.6 g/100 g edible portion for *Ekwang* and was significantly different (*P *<* *0.05). The amount of moisture is dependent on the type of dish and also the amount of water used in the preparation. The moisture content was higher compared to the value (50 g/100 g edible portion) found by Okeke and Eze (2006) in *Akara* (traditional dish) prepared from Bean cake and consumed in Nsukka Nigeria. But lower than moisture content of 82.4 g/100 g edible portion reported by Jaarsveld et al. (2014) in cowpea.

**Table 2:** Proximate, mineral, carotenoid, and vitamin A activity compositions per 100 g edible portion of dishes

Parameters	Unit	*Ekomba*	*Ekwang*	*Tenue militaire*	*Koki*
Proximate composition					
Moisture	g	61.5 ± 3.5^c^	77.6 ± 4.8^a^	76.4 ± 5.2^a^	71.0 ± 4.4^b^
Ash	g	1.6 ± 0.1^a^	1.1 ± 0.1^a^	1.0 ± 0.1^a^	1.7 ± 0.1^a^
Protein	g	5.0 ± 0.5^a^	1.4 ± 0.1^b^	2.1 ± 0.2^b^	5.4 ± 0.2^a^
Fat	g	8.0 ± 1.4^a^	4.3 ± 0.6^b^	5.3 ± 0.2^b^	5.9 ± 0.3^b^
Dietary fiber	g	1.7 ± 0.4^a^	0.2 ± 0.1^d^	0.8 ± 0.1^c^	0.9 ± 0.1^b^
Carbohydrates	g	22.2 ± 3.2^a^	15.4 ± 1.3^b^	14.4 ± 1.7^b^	15.2 ± 0.3^b^
Energy	Kcal	180.8 ± 6.4^a^	105.7 ± 5.3^d^	113.5 ± 6.4^c^	135.2 ± 2.1^b^
Minerals					
Calcium	mg	30.4 ± 1.4^b^	13.4 ± 1.7^d^	24.4 ± 0.4^c^	38.9 ± 0.3^a^
Magnesium	mg	30.7 ± 1.7^a^	12.9 ± 1.4^d^	17.3 ± 0.8^c^	24.5 ± 3.2^b^
Sodium	mg	567.9 ± 3.4^a^	336.2 ± 2.1^d^	375.1 ± 3.9^c^	516.4 ± 5.1^b^
Potassium	mg	96.7 ± 4.8^c^	127.0 ± 2.3^b^	63.3 ± 4.3^d^	182.7 ± 3.2^a^
Iron	mg	0.5 ± 0.1^d^	1.2 ± 0.1^c^	1.5 ± 0.3^a^	1.4 ± 0.6^b^
Zinc	mg	0.6 ± 0.1^b^	0.3 ± 0.1^c^	0.3 ± 0.1^c^	1.1 ± 0.1^a^
Copper	mg	0.1 ± 0.0^b^	0.2 ± 0.0^a^	0.2 ± 0.0^a^	0.1 ± 0.0^b^
Manganese	mg	0.4 ± 0.1^c^	0.3 ± 0.1^d^	0.4 ± 0.1^a^	0.4 ± 0.1^b^
Carotenoids and vitamin A					
Lutein	mg	0.3 ± 0.0^c^	1.6 ± 0.2^a^	1.4 ± 0.1^b^	0.4 ± 0.1^c^
*β*‐carotene	mg	1.3 ± 0.1^b^	3.9 ± 0.1^a^	4.3 ± 0.3^a^	4.3 ± 0.2^a^
Vitamin A activity	mg RAE	0.1 ± 0.0^b^	0.3 ± 0.0^a^	0.4 ± 0.1^a^	0.4 ± 0.1^a^

Results are the means ±SD of six samples with three replicates per variety. Mean values with different superscript letters in the same row are significantly different (*P *<* *0.05).

RAE, Retinol activity equivalents.

The ash content in the samples was in the range of 1.0 (*Tenue militaire*) to 1.7 g/100 g edible portion (*Koki*). These values were not significantly different (*P *>* *0.05) and were lower than ash content of 1.76 g/100 g edible in cowpea (Jaarsveld et al. 2014).

The protein content in the samples varied from 1.4 for *Ekwang* to 5.4 g/100 g edible portion for *Koki* and was significantly different (*P *<* *0.05). These variations can be attributed to the type and quantity of ingredients used. Peanut and cowpea seeds increased the protein content of the dishes. The protein content in the samples studied was higher than the protein content of 0.8 g/100 g edible portion reported by Sharma et al. (2007) in Pap (hot cereal) consumed in the Centre Region of Cameroon, but had a lower protein content of 11.2 g/100 g edible portion reported by Caidan et al. (2014) in the fruit. The nutritional quality of *Ekomba* and *Koki* will improve the nutritional status of the population based on its protein content.

The fat content in the samples ranged from 4.3 for *Ekwang* to 8.0 g/100 g edible portion for *Ekomba* and was significantly different (*P *<* *0.05). These values were higher in fat content of 0.4 g/100 g edible portion in *Akamu* (maize gruel) consumed in Nsukka Nigeria (Okeke and Eze 2006). The higher fat content of the samples studied was due to the addition of peanut and crude palm oil.

The dietary fiber content in the samples was in the range of 0.2 to 1.7 g/100 g edible portion. The lowest dietary fiber content was obtained from *Ekwang* while the highest was obtained from *Ekomba*. The values of the dietary fiber content of collected samples were significantly different (*P *<* *0.05). The dietary fiber content of the samples was similar to the values (0.26–1.69 g/100 g edible portion) found by Okeke et al. (2009) in traditional foods consumed in Igbo‐Nigeria.

The carbohydrate content in the samples ranged between 14.4 for *Tenue militaire* to 22.2 g/100 g edible portion for *Ekomba*. The values of the carbohydrate content in the collected samples were significantly different (*P *<* *0.05). The carbohydrate content of the samples had a lower carbohydrate content of 34.02 g/100 g edible portion in *Ayaraya Oka* (steamed maize pudding with vegetable) consumed in Igbo‐Nigeria (Okeke et al. 2009) and 39.4 g/100 g edible portion found in Fufu corn, consumed in Yaoundé Centre‐Cameroon (Sharma et al. 2007).

The energy content in the samples varied from 105.9 for *Ekwang* to 180.8 Kcal/100 g edible portion for *Ekomba* and was significantly different (*P *<* *0.05). The energy content of the samples was higher than the value of 77 Kcal/100 g edible portion found by Spearing et al. (2012) and consumed in South Africa.

### Mineral composition

Table 2 also presents the content of the mineral analysis.

Sodium and potassium could be considered as the majors minerals in samples. Sodium and potassium contents ranged from 336.2 (*Ekwang*) to 567.9^ ^mg/100 g edible portion (*Ekomba*) and 63.3 (*Tenue militaire*) to 182.7^ ^mg/100 g edible portion (*Koki*), respectively. Other minerals and their concentration in samples were calcium, 13.4 (*Ekwang*) to 38.9^ ^mg/100 g edible portion (*Koki*); magnesium, 12.9 (*Ekwang*) to 30.7 mg/100 g edible portion (*Ekomba*); iron, 0.5 (*Ekomba*) to 1.5 mg/100 g edible portion (*Tenue militaire*); zinc, 0.3 (*Ekwang*) to 1.1 mg/100 g edible portion (*Koki*) and manganese, 0.3 (*Ekwang*) to 0.4 mg/100 g edible portion (*Tenue militaire*). Copper was the least abundant mineral element in samples with concentration range of 0.1 (*Ekomba* and *Koki*) to 0.2 mg/100 g edible portion (*Ekwang*). A significant difference was observed in the distribution of all the minerals (*P *<* *0.05).

The sodium content in all the samples (>300 mg/100 g edible portion) was higher than the sodium content of 119.19 mg/100 g edible portion in cereals and cereal products consumed in Yaoundé‐Cameroon (Gimou et al. 2014). Furthermore, potassium content of 63.3–182.7 mg/100 g edible portion in samples was higher than the potassium content of 14.6 mg/100 g edible portion found by Sharma et al. (2007) in Pap (hot cereal) consumed in Yaoundé‐Cameroon. Calcium content in samples (13.4–38.9 mg/100 g edible portion) was higher than the calcium content of 10.89 mg/100 g edible portion found in *Ewedu* (traditional dish) consumed in Nigeria (Kayode et al. 2010). Calcium content in samples was also higher than the calcium content of 4 mg/100 g edible portion found in *Putu* (traditional refined maize) consumed in South Africa (Spearing et al. 2012). More ever, the magnesium content of samples (12.9–30.7 mg/100 g edible portion) was higher than the magnesium content of 4.8 mg/100 g edible portion found in boiled rice (Gimou et al. 2014). The iron content in samples (0.5–1.5 mg/100 g edible portion) was higher than the iron content of (0.22–0.32 mg/100 g edible portion) found in traditional dishes consumed in Igbo‐Nigeria (Okeke and Eze 2006). Iron content in samples was also higher than the iron content of 0.17 mg/100 g edible portion found by Dashti et al. (2004) in *Khathra* (mixed fish and rice) consumed in Kuwait. Zinc content in samples (0.3–1.1 mg/100 g edible portion) was higher than the zinc content of 0.19 mg/100 g edible portion found in *Ramirebaka* (traditional dish) consumed in an urban district of Antananarivo in Madagascar (Randrianatoandro et al. 2010).

The manganese content in the samples (0.3–0.4 mg/100 g edible portion) was similar to the manganese content of 0.33 mg/100 g edible portion in cereal and cereal products consumed in Yaoundé (Gimou et al. 2014). Copper content in samples (0.1–0.2 mg/100 g edible portion) was similar to the copper content of 0.12 mg/100 g edible portion in tubers and tuber products consumed in Yaoundé (Gimou et al. 2014).

### Carotenoid and vitamin A activity compositions

The carotenoids identified in the samples were lutein and *β*‐carotene (Table 2). All the samples were good carotenoid sources. *Ekwang* had the highest amount of lutein (1.6 mg/100 g edible portion). *Ekawang, Tenue militaire,* and *Koki* had the highest amount of *β*‐carotene (3.9, 4.3 and 4.3 mg/100 g edible portion), respectively. The higher level of *β*‐carotene in samples could be invariably attributed to the use of crude red palm oil, which is one of the richest sources of *β*‐carotene for the preparation of these dishes. Important health benefits have been attributed to the carotenoids. Some carotenoids, such as *β*‐carotene, are capable of being converted into vitamin A, thereby they play an important nutritional role.

All the samples were rich in vitamin A activity composition. The values obtained (0.1–0.4 mg RAE/100 g edible portion) were higher compared to (0.06–0.09 mg RAE/100 g edible portion) found by Ponka et al.(2007) in some traditional sauces consumed in a village situated in the Central Region of Cameroon called Ngali II.

Contribution of 100 g edible portion of dishes to dietary reference intakes (%) for children aged 1–2 years.

Contribution of dietary reference intakes (%) for children aged 1–2 years for macronutrients energy, minerals and vitamin A are presented in the Table 3. Consumption of 100 g of each dish would meet 10.8–41.6%, 14.2–26.6%, 1.1–9.0%, 11.1–17.1% and 11.1–17.1% of the dietary reference intakes, respectively, for protein, fat, dietary fiber, carbohydrates, and energy. These values were higher compared to the values obtained from five varieties of pap commonly consumed in Maroua‐Cameroon (Ponka et al. 2015; a). On the other hand, consumption of 100 g of each dish would meet 2.7–7.8%, 16.2–38.4%, 33.6–56.8%, 2.1–6.1%, 7.2–21.6%, 8.9–37.8%, 36.228–55.9%, and 23.3–34.4% of the dietary reference intakes, respectively, for calcium, magnesium, sodium, potassium, iron, zinc, copper, and manganese. These values were also higher compared to the values obtained from five varieties of pap commonly consumed in Maroua‐Cameroon (Ponka et al. 2015).

**Table 3:** Contribution of 100 g edible portion of dishes to dietary reference intakes (%) for children aged 1–2 years

Parameters	DRI^a^	*Ekomba*	*Ekwang*	*Tenue militaire*	*Koki*
Protein	13 g/day	38.2	10.8	16.4	41.6
Fat	30 g/day	26.6	14.2	17.5	19.5
Dietary fiber	19 g/day	9.0	1.1	4.1	4.9
Carbohydrates	130 g/day	17.1	11.9	11.1	11.7
Energy	1350 cal/day	13.4	7.8	8.4	10.0
Calcium	500 mg/day	6.1	2.7	4.9	7.8
Magnesium	80 mg /day	38.4	16.2	21.6	30.6
Sodium	1000 mg/day	56.8	33.6	37.5	51.6
Potassium	3000 mg/day	3.2	4.2	2.1	6.1
Iron	7 mg/day	7.2	16.6	21.6	19.4
Zinc	3 mg/day	21.2	8.9	10.6	37.8
Copper	0.34 mg/day	36.2	55.9	49.9	36.7
Manganese	1.2 mg/day	29.5	23.3	34.4	31.5
Vitamin A	0.3 mg RAE/day	35.9	108.8	119.2	119.1

a Dietary reference intakes (DRI) established by the Food and Nutrition Board of the Institute of Medicine (IOM) (Institute of Medicine 2000, 2001, & 2004); and the Food and Nutrition Board of the National Research Council (NRC) (2005). Values given are for children aged 1–2 years.

### Amino acid profile

Table 4 shows the amino acid profiles of the samples. Except methionine, significant difference was observed in the distribution of all the amino acid among all the samples (*P *<* *0.05). Leucine was the most abundant occurring essential amino acid in the samples studied, while glumatic acid was the most abundant nonessential amino acid in all the samples tested. *Koki* had the highest concentration of these amino acids with 76.4 mg leucine/g protein and 157.6 mg glutamic acid/g protein. These observations corroborated by earlier reported trends of leucine and glutamic acid being the most concentrated essential and nonessential amino acids, respectively, in some pap commonly consumed in Maroua‐Cameroon (Ponka et al. 2015).

**Table 4:** Amino acids composition (mg/g protein) of dishes

Amino acids	*Ekomba*	*Ekwang*	*Tenue militaire*	*Koki*
Essential amino acids				
His	19.0 ± 0.7^b^	13.4 ± 0.9^d^	15.5 ± 1.2^c^	28.6 ± 0.8^a^
Thr	28.0 ± 1.1^c^	33.0 ± 0.8^b^	25.2 ± 0.5^d^	44.0 ± 0.7^a^
Val	37.6 ± 1.4^c^	46.7 ± 0.7^a^	32.3 ± 1.9^d^	39.8 ± 0.8^b^
Met	0.9 ± 0.1^a^	1.3 ± 0.3^a^	1.6 ± 0.7^a^	1.6 ± 0.4^a^
Ile	29.5 ± 1.4^b^	26.7 ± 0.4^c^	22.8 ± 1.3^d^	41.7 ± 1.7^a^
Leu	69.9 ± 0.3^b^	53.9 ± 0.8^c^	74.6 ± 0.3^a^	76.4 ± 0.3^a^
Phe	42.2 ± 1.7^b^	42.9 ± 0.5^b^	31.0 ± 0.2^c^	55.9 ± 1.7^a^
Lys	19.1 ± 0.3^c^	34.9 ± 0.7^b^	16.7 ± 0.4^d^	60.5 ± 1.4^a^
TEAA	246.2 ± 20.3	252.8 ± 17.5	219.6 ± 21.4	348.5 ± 22.4
Nonessential amino acids				
Asp	84.1 ± 1.5^c^	89.9 ± 1.1^b^	45.7 ± 1.6^d^	112.9 ± 2.3^a^
Glu	149.3 ± 1.9^b^	122.7 ± 1.3^c^	158.7 ± 1.5^a^	157.6 ± 2.1^a^
Ser	36.6 ± 1.9^b^	38.4 ± 1.3^b^	27.0 ± 0.8^c^	44.0 ± 1.9^a^
Gly	43.8 ± 1.1^b^	48.0 ± 1.6^a^	23.5 ± 0.8^d^	35.2 ± 0.6^c^
Arg	73.1 ± 0.8^a^	58.4 ± 0.4^c^	33.5 ± 0.2^d^	68.0 ± 0.4^b^
Ala	42.5 ± 0.6^c^	45.0 ± 0.8^b^	47.6 ± 0.9^a^	39.8 ± 0.3^d^
Pro	47.7 ± 0.8^b^	33.8 ± 1.1^d^	52.0 ± 0.9^a^	38.2 ± 0.3^c^
Tyr	30.7 ± 0.5^a^	24.8 ± 0.2^b^	21.8 ± 0.2^c^	30.0 ± 0.8^a^
Cys	8.1 ± 0.7^a^	5.4 ± 0.3^b^	5.4 ± 0.6^b^	5.7 ± 0.4^b^
TNEAA	515.9 ± 41.0	466.4 ± 35.3	415.2 ± 44.7	531.5 ± 47.4
TAA	762.1	719.2	634.7	880.0
∑TEAA / ∑TAA (%)	32.3	35.2	34.6	39.6

Results are the means ±SD of six samples with three replicates per variety; Mean followed by different superscript letters in the same row are significantly different (*P *<* *0.05).

His, Histidine; Thr, Threonine; Val, Valine; Met, Methionine; Ile, Isoleucine; Leu, Leucine; Phe, Phenylalanine; Lys, Lysine; Asp, Aspartic acid; Glu, Glutamic acid; Ser, Serine; Gly, Glycine; Arg, Arginine; Ala, Alanine; Pro, proline; Tyr, Tyrosine; Cys, Cysteine; TEAA, Total Essentiel amino acids; TNEAA, Total Nonessential amino acids; TAA, Total amino acids.

This amino acid profile was similar to the amino profile of food consumed in Northwest Mexico reported by Caire‐Juvera et al. (2013). The nutritive value of a protein depends primarily on the capacity to satisfy the needs for nitrogen and essential amino acids (Salunkhe et al. 1985). All essential amino acids are found to be present in these samples investigated except tryptophan, which was not determined. Except in *Ekomba*, essential amino acids represented up to 33% of total amino acids in all samples*,* which according to Blankership and Alford (1983), indicated a good equilibrium between amino acid.

Essential amino acid composition (mg/g protein) of dish compared to FAO/WHO/UNU (2007) reference pattern suggested for children aged 1–2 years in mg/g protein.

In Table 5, the essential amino acid composition (mg/g protein) of the samples was compared to the reference levels required by children in mg/g protein recommended by FAO/WHO/UNU (2007) for children of 1–2 years old in mg/g protein. In fact, the body is unable to synthesize essential amino acids and children 1–2 years need the most of these amino acids for their development. The histidine in all samples met the recommended children requirement (18 mg/g protein) except in *Ekwang and Tenue militaire*. The threonine in all sauce samples met the recommended children requirement (27 mg/g protein) except in *Tenue miliatire*. The valine met the recommended requirement for children (41 mg/g protein) only in *Ekwang*. The combination of methionine and cysteine in all samples was lower than the recommended value (25 mg/g protein). The isoleucine met the recommended requirement for children (31 mg/g protein) only in *Koki*. The leucine in all samples met the recommended requirement for children (63 mg/g protein) except in *Ekwang*. The combination of phenylalanine and tyrosine in all sauce samples met the recommended requirement for children (46 mg/g protein); the lysine met the recommended children requirement (52 mg/g protein) only in *Koki*.

**Table 5:** Essential amino acids composition (mg/g protein) of dishes compared to FAO/WHO/UNU (2007) reference pattern suggests for children aged 1–2 years in mg/g protein

Amino acids	*Ekomba*	*Ekwang*	*Tenue militaire*	*Koki*	FAO/WHO/UNU (2007) 1–2 years
His	19.0	13.4	15.5	28.6	18
Thr	28.0	33.0	25.2	44.0	27
Val	37.6	46.7	32.3	39.8	41
Met+Cys	9.1	6.7	7.0	7.3	25
Ile	29.5	26.7	22.8	41.7	31
Leu	69.9	53.9	74.6	76.4	63
Phe+Tyr	72.9	67.7	52.8	85.9	46
Lys	19.1	34.9	16.7	60.5	52

His, Histidine; Thr, Threonine; Val, Valine; Met+Cys: Methionine+Cysteine; Ile, Isoleucine; Leu, Leucine; Phe+Tyr, Phenylalanine+ Tyrosine; Lys, Lysine.

Amino acid score (%) of dish based on the essential amino acid content and the pattern for children aged 1–2 years FAO/WHO/UNU (2007).

Table 6 shows the amino acid scores (%) for the samples analyzed in this study based on the essential amino acid content and the pattern for children between 1–2 years old following the FAO/WHO/UNU (2007) recommendation. In each sample amino acid with a lower amino acid score (%) represents the limiting amino acid for the sample. As expected, the methionine and cysteine were the first limiting amino acid in all samples followed by lysine except in *Koki* in which the second limiting amino acid was leucine. These observations are in agreement with a previous report of methionine + cysteine being the first limiting amino acid in *Onunu* and *Mgbam*, traditional dishes of the Ikwerre people of Nigeria (Amadi et al. 2011).

**Table 6:** Amino acid score (%) of dishes based on the essential amino acid content and the pattern for children aged 1–2 years FAO/WHO/UNU (2007)

Amino acids	*Ekomba*	*Ekwang*	*Tenue militaire*	*Koki*
His	105.4	74.7	86.1	158.9
Thr	103.6	122.1	93.4	163.0
Val	91.6	114.0	78.8	97.2^b^
Met+Cys	36.2^a^	26.9^a^	27.9^a^	29.0^a^
Ile	95.2	86.2	73.5	134.6
Leu	110.9	85.6	118.4	121.3
Phe+Tyr	158.5	147.1	114.7	186.7
Lys	36.8^b^	67.1^b^	32.1^b^	116.3

His, Histidine; Thr, Threonine; Val, Valine; Met+Cys, Methionine+Cysteine; Ile, Isoleucine; Leu, Leucine; Phe+Tyr, Phenylalanine+ Tyrosine; Lys, Lysine.

a Frist limiting amino acid;

b Second limiting amino acid.

## Conclusion

We have provided the nutrient content of some traditional dishes consumed in Yaoundé‐Cameroon. The results suggest that *Ekomba* and *Koki* have the highest content of protein. All dishes contained a substantial amount of fat and minerals. *Ekwang*,* Tenue militaire*, and *Koki* are rich in vitamin A activity. Consumption of each dish (100 g) (*Ekwang*,* Tenue militaire*, and *Koki*) by children aged 1–2 years would meet more than 100% of their daily recommended intake for vitamin A which is a micronutrient of public health significance in Cameroon. All dishes present a good equilibrium between amino acid except in Ekomba. This up‐to‐date appropriate information will contribute to the calculation of accurate energy and nutrient intake for this population, and should be used to encourage the consumption of dishes rich in key nutrients.

## Conflict of Interest

None declared.
